# Ultra-High-Resolution CT of the Head and Neck with Deep Learning Reconstruction—Assessment of Image Quality and Radiation Exposure and Intraindividual Comparison with Normal-Resolution CT

**DOI:** 10.3390/diagnostics13091534

**Published:** 2023-04-24

**Authors:** Sebastian Altmann, Mario A. Abello Mercado, Felix A. Ucar, Andrea Kronfeld, Bilal Al-Nawas, Anirban Mukhopadhyay, Christian Booz, Marc A. Brockmann, Ahmed E. Othman

**Affiliations:** 1 Department of Neuroradiology, University Medical Center Mainz, Johannes Gutenberg University, Langenbeckst. 1, 55131 Mainz, Germany; sebastian.altmann@unimedizin-mainz.de (S.A.); mario.abello@unimedizin-mainz.de (M.A.A.M.); felixanton.ucar@unimedizin-mainz.de (F.A.U.); andrea.kronfeld@unimedizin-mainz.de (A.K.); Brockmann@gmx.de (M.A.B.); 2 Department of Oral and Maxillofacial Surgery, University Medical Center Mainz, Johannes Gutenberg University, Langenbeckst. 1, 55131 Mainz, Germany; bilal.al-nawas@unimedizin-mainz.de; 3 Department of Computer Science, Technical University of Darmstadt, Fraunhoferst. 5, 64283 Darmstadt, Germany; anirban.mukhopadhyay@gris.informatik.tu-darmstadt.de; 4 Department of Diagnostic and Interventional Radiology, University Clinic Frankfurt, Theodor-Stern-Kai 7, 60590 Frankfurt, Germany; christian.booz@kgu.de

**Keywords:** computed tomography, head and neck neoplasms, ultra-high resolution, image quality, radiation dose, deep learning

## Abstract

Objectives: To assess the benefits of ultra-high-resolution CT (UHR-CT) with deep learning–based image reconstruction engine (AiCE) regarding image quality and radiation dose and intraindividually compare it to normal-resolution CT (NR-CT). Methods: Forty consecutive patients with head and neck UHR-CT with AiCE for diagnosed head and neck malignancies and available prior NR-CT of a different scanner were retrospectively evaluated. Two readers evaluated subjective image quality using a 5-point Likert scale regarding image noise, image sharpness, artifacts, diagnostic acceptability, and assessability of various anatomic regions. For reproducibility, inter-reader agreement was analyzed. Furthermore, signal-to-noise ratio (SNR), contrast-to-noise ratio (CNR), and slope of the gray-value transition between different tissues were calculated. Radiation dose was evaluated by comparing CTDIvol, DLP, and mean effective dose values. Results: UHR-CT with AiCE reconstruction led to significant improvement in subjective (image noise and diagnostic acceptability: *p* < 0.000; ICC ≥ 0.91) and objective image quality (SNR: *p* < 0.000; CNR: *p* < 0.025) at significantly lower radiation doses (NR-CT 2.03 ± 0.14 mSv; UHR-CT 1.45 ± 0.11 mSv; *p* < 0.0001) compared to NR-CT. Conclusions: Compared to NR-CT, UHR-CT combined with AiCE provides superior image quality at a markedly lower radiation dose. With improved soft tissue assessment and potentially improved tumor detection, UHR-CT may add further value to the role of CT in the assessment of head and neck pathologies.

## 1. Introduction

Computed tomography (CT) is indicated for precise tumor staging and follow-up examinations in patients suffering from head and neck neoplasia. As affected structures of the head and neck region, as well as the skull base, are usually quite subtle, high resolution is important for accurate tumor evaluation. Furthermore, as the depth of invasion has become an important aspect in the staging of oropharyngeal cancer, the exact assessment of the tumor extension is of increasing importance in local tumor staging [1].

Although magnetic resonance imaging (MRI) is superior to CT with regard to soft tissue contrast and is the method of choice, especially in younger patients, due to its radiation-free technique, a potential disadvantage of MRI is its comparatively long examination time. Thus, sufficient patient compliance is imperative. On the other hand, CT is widely available, more cost-effective, and enables excellent imaging of bony structures [2,3]. Patients suffering from head and neck tumors are often older, frequently pre-diseased, and often present with an impaired general condition. Thus, in order to generate conclusive diagnostic images of high quality, the minimization of motion artifacts in these patients and consequently short examination times are very important. Therefore, CT is typically the first-line imaging tool for initial tumor staging as well as for urgent evaluation of infectious diseases.The constant technological progress with regard to software (e.g., iterative reconstruction (IR)) and hardware development has enabled a continuous improvement in image quality, an increased diagnostic confidence, and a reduction in the required radiation dose [4,5,6,7]. With these technological advances, an ultra-high-resolution CT (UHR-CT) with a focal spot size of 0.4 mm × 0.5 mm, detector elements with 0.25 × 0.25 mm, a beam collimation of 0.25 mm × 160 mm rows, and a slice thickness of 0.25 mm have been presented and implemented for clinical routine. It will thereby enable ultra-high image quality and improve the detectability of even very small pathologies and contour irregularities with a spatial resolution up to 150 µm. 

An increase in spatial resolution is associated with an increase in image noise; therefore, higher radiation doses are required to maintain low-contrast detectability [8]. To counteract the increase in radiation dose, advanced image reconstruction algorithms may be used to reduce image noise. Recently, a new deep-learning reconstruction technique, Advanced intelligent Clear-IQ Engine (AiCE), has been developed to further optimize image quality [9,10,11].

No published data regarding the usefulness of UHR-CT in the evaluation of head and neck malignancies exist to date. We hypothesized that UHR-CT in combination with deep-learning image reconstruction leads to superior image quality at lower radiation dose levels in head and neck imaging compared to normal-resolution CT (NR-CT). As the survival rate of head and neck pathologies is steadily increasing, reduction in radiation exposure becomes increasingly important. We therefore aimed for the first time to intraindividually compare image quality and radiation dose of a UHR-CT of the head and neck combined with AiCE to NR-CT in patients with clinically diagnosed head and neck malignancies. 

## 2. Materials and Methods

This retrospective study was approved by the Ethics Committee of the Rhineland-Palatinate Chamber of Physicians, and written informed consent was waived along with the ethical approval number 2021-15948_2 and the approval date 2 December 2022. The study was conducted in accordance with the Helsinki Declaration as revised in 2013. 

### 2.1. Patient Cohort

**Figure 1:**
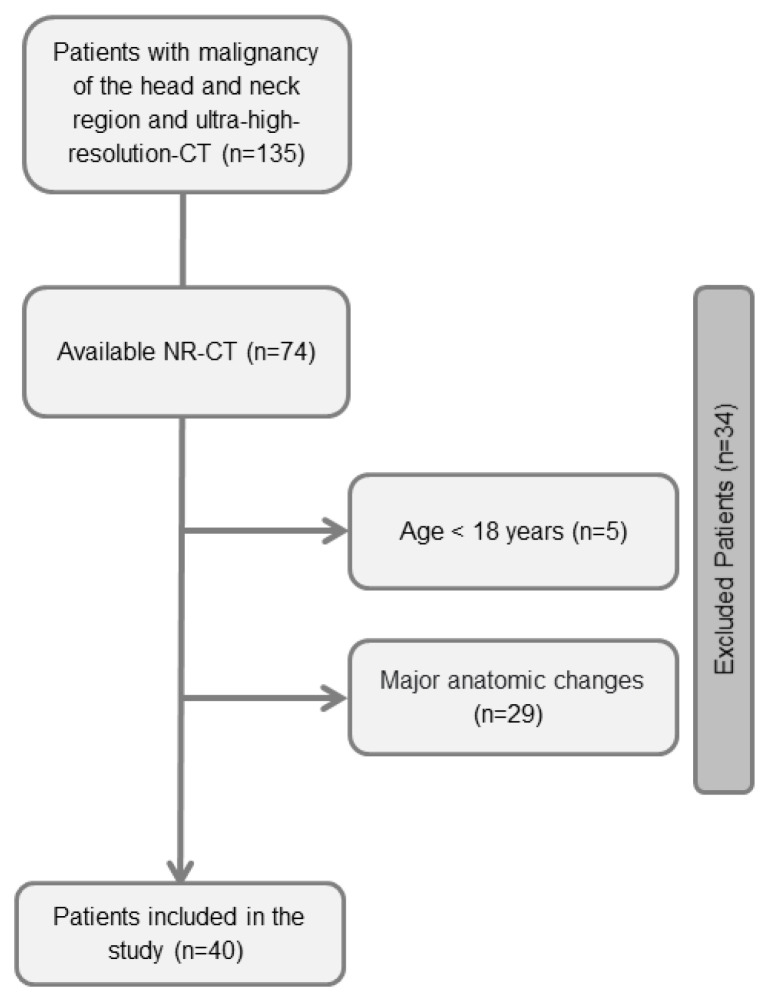
Flow chart of the inclusion/exclusion process.

During the inclusion period between March 2021 and October 2021, 135 consecutive patients underwent UHR-CT of the head and neck. The inclusion criteria were (i) known head and neck neoplasia and (ii) prior examination with NR-CT within 2 years. The exclusion criteria were (i) age younger than 18 years, (ii) non-contrast CT studies, (iii) major anatomic changes between both CT examinations (e.g., extensive surgery with postoperative dental prosthesis and broad beam hardening artifacts), and (iv) major soft tissue alterations (e.g., distinct anasarca and interstitial edema after radiotherapy). The inclusion/exclusion process is presented in Figure 1.

### 2.2. Image Acquisition

UHR-CT images were acquired using an Aquilion Precision scanner (Canon Medical Systems) CE-certified scanner, with a focal spot size of 0.4 mm × 0.5 mm (smallest), detector elements with 0.25 × 0.25 mm, a slice thickness of 0.25 mm, a reconstruction matrix of 1024 × 1024, and a beam collimation of 0.25 mm × 160 rows with 1792 channels. The CT images were acquired with a tube voltage of 120 kV, a spiral pitch factor of 0.569, a field view (FoV) of 240 mm, and a rotation time of 0.5 s per rotation. The data was reconstructed with body kernel and an ultra-high-resolution deep learning–based algorithm AiCE with a matrix of 1024. Thereby the data was reconstructed in coronal, transversal, and sagittal view, using a slice thickness of 1 mm and 3 mm. 

NR-CT images were acquired using an Aquilion 32 scanner (Toshiba Medical Systems) with a matrix of 512 × 512 and a focal spot size 1.6 × 1.4. The scanner featured a detector element size of 0.5 mm, a beam collimation of 0.5 mm × 32 rows, and a spatial resolution of 18 lp/cm. The helical-CT parameters of the neck protocol consisted of a focal spot size of 0.8 × 1.3 mm and a tube voltage of 120 KV, a field of view of 240 mm, a rotation time of 0.5 s per rotation, and a pitch of 0.8. The data was reconstructed using a slice thickness of 1 mm and 3 mm, together with a reconstruction kernel of 04.

Both CT scanners utilized auto exposure control (AEC) automated current adjustment mode. The applied contrast protocol was similar for both examinations. Contrast injection was conducted through a high-pressure syringe system for advanced clinical CT imaging procedures (Accutron CT-D; Medtron) using a nonionic contrast agent (iopromide, Ultravist-370; Bayer Healthcare) via an 18G peripheral venous catheter placed in the cubital vein. A total of 110 mL Ultravist 370 was injected, in accordance with our specific protocol for imaging of the head and neck region. Thereby, at first 65 mL of the contrast agent was injected at a flow rate of 1.5 mL per second (injection time 44 s), immediately followed by a 25 mL saline bolus (flow rate 2.0 mL/s; injection time 12 s) and a second 10 mL saline bolus (flow rate 0.1 mL/injection time 100 s). Thereafter a second contrast bolus of 50 mL was administered (flow rate 5 mL/injection time 17 s). The CT scan automatically started 180 s after the start of the injection. The technical parameters of both CT scanners are compared in Table 1.

### 2.3. Subjective Image Evaluation

The subjective image quality was assessed by two board-certified radiologists, both with at least 5 years of experience in head and neck imaging (S.A.; M.A.A.M.). Both readers were briefed, and exemplary cases were demonstrated to attain consensus and standardization on how to apply a 5-point Likert scale. 

The raters assessed the image noise, image sharpness, artifacts, and diagnostic acceptability of the images. Furthermore, the assessability of the following anatomic regions was also evaluated by both raters: skull base, infratemporal fossa, nasal cavity, paranasal sinuses, nasopharyngeal space, oropharyngeal space, hypopharyngeal space, oral cavity and buccal mucosa, floor of mouth, lymph nodes level I, lymph nodes level II-IV, jugular fossa, thyroid and upper mediastinum, salivary glands, and the carotid and vertebral arteries separated into three anatomic sections: from the vascular origin to the carotid bifurcation, the carotid bifurcation itself, and from the bifurcation to the skull base. 

Image quality was based on the adapted guidelines of the European guidelines on quality criteria for CT [12,13]. The 5-point Likert scale was consistently used throughout all patients and categories, except for artifacts through foreign materials, where a 4-point Likert scale was used (Table 2). As UHR CT is performed in clinical routine at our institution, this image quality was declared as the gold standard. Readers were free to use 1 mm or 3 mm slices. In order to reduce recall bias, NR-CT and UHR-CT images were mixed and randomized and evaluated six weeks later. Notably, the image annotations were blinded, and both readers were uninformed that for each patient both NR-CT and UHR-CT images were included.

### 2.4. Objective Image Evaluation

**Figure 2:**
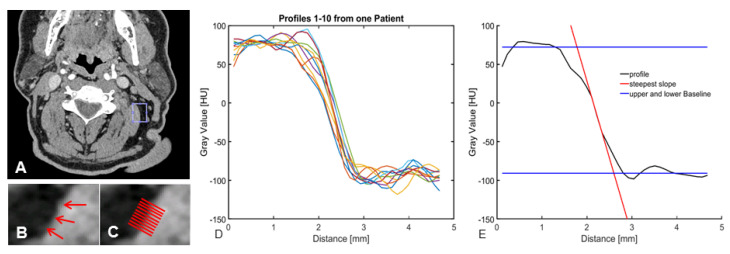
Individual steps of objective image evaluation, (**A**) sliding window of 5 mm × 5 mm, (**B**) red arrows display the selected interface, (**C**) each red line represents a perpendicular profile, (**D**) graphical illustration of the 10 perpendicular profiles, (**E**) graphical illustration of measurements for edge sharpness.

To evaluate objective image parameters, signal-to-noise ratio (SNR, muscle), contrast- to-noise ratio (CNR, muscle, and fat), and the slope of the gray-value transition from fat to muscle tissue as measures of image sharpness were used for an observer-independent description of the image quality. A 3 mm reconstruction was used to determine exemplary slices, selected by a radiologist with 5 years of training, focusing on the cervical soft tissues at the level of the mandible in the proximity of the sternocleidomastoid muscle. On the chosen slice, a small region was selected and the scope of the borderline, which should be analyzed, was defined. Ten profiles perpendicular to the marking were determined over a range of 4.5 mm to each side. As demonstrated in Figure 2, for each of the profiles, the upper and the lower baseline, as well as the steepest slope of the transition over three points, as a measure for edge sharpness, were detected. Furthermore, the median was calculated for all ten profiles.

Noise determination was designed to find the greatest possible homogeneous region in striking distance to the evaluated profiles. Hence, a small region of 5 mm × 5 mm, without any sharp changes in gray values as induced by tissue borders in the surrounding tissue, was identified. The eligible region with the lowest variation in gray values (i.e., with lowest standard deviation) was chosen, to eliminate further sources of gray value variation except noise.

**Figure 3:**
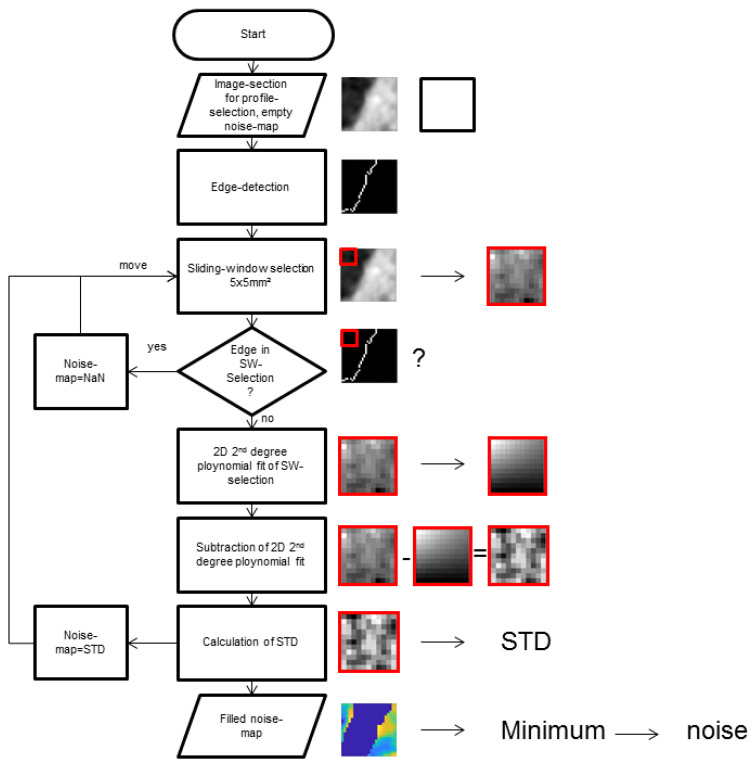
Flow chart of noise calculation.

Noise distribution was determined in three steps: Firstly, the position of transition from high to low signal intensities was detected by an edge detection algorithm [14]. Secondly, a sliding window of 5 mm × 5 mm was applied. In case the area did not include the previously determined edge position, a second-order 2D-polynomial function was fitted. These results were subtracted from the initial gray values in order to eliminate low frequency drifts in gray values [15]. Finally, the standard deviation of the gray values in the sliding window of 5 mm × 5 mm was calculated and written to a noise map. Noise was assumed to be the smallest value in the resulting parameter map [14,16]. The SNR was calculated as the signal intensity of the upper baseline divided by the noise value, and the CNR was calculated as the difference in signal intensity of the upper and lower baseline divided by the noise value. The flowchart for noise calculation is shown in Figure 3.

A similar evaluation of the objective image quality has been performed previously, although to some extent, we applied minor changes to ensure optimal image evaluation of this specific anatomic region [14,16,17,18,19]. 

### 2.5. Radiation Dose

To access the estimated radiation dose, we evaluated descriptors including computed tomography dose index (CTDIvol) and scan length, as well as DLP as reported by the CT system. The mean scan length of all the CT scans was 24.85 cm [24.03–25.65]. For comparability, the DLP values were normalized according to the approximated mean scan length of 25 cm. The effective dose values were calculated, multiplying the normalized dose length product with the International Commission on Radiological Protection conversion factor for head and neck CT (k = 0.0058) [20]. 

### 2.6. Statistical Analysis 

Statistical analysis was performed using SPSS (SPSS IBM Statistics for Windows, Version 23.0 IBM Corp). Continuous variables were reported as mean ± standard deviation if normally distributed, and as median/interquartile range in case of non-normal distribution. Categorical variables were displayed as absolute frequencies and proportions. The Kolmogorov–Smirnov test was used to assess normal distribution of the continuous data. Mean, median, and standard deviation as well as interrater agreement (Cohen’s kappa coefficient) for continuous variables were calculated. Intraclass correlation (ICC) was determined with two-way mixed effects and focused on consistency. The level of agreement was defined as follows: poor, ICC < 0.5; moderate, ICC = 0.5–0.75; good, ICC = 0.76–0.9; excellent, ICC > 0.9 [21]. The Wilcoxon–Mann–Whitney test was applied for non-parametric categorical variables, and the t-test was applied for continuous variables. *p*-values less than 0.05 were considered statistically significant.

## 3. Results

### 3.1. Patient Cohort

The final study sample consisted of 40 patients (22 men and 18 women), with a mean age of 65 years (age range between 19–89 years). Diagnosis included squamous cell carcinoma of the tongue (*n* = 16), squamous cell carcinoma of the lower jaw (*n* = 7), squamous cell carcinoma of the floor of mouth (*n* = 6), squamous cell carcinoma of the cheek (*n* = 2), giant cell tumor (*n* = 2), mucoepidermoid carcinoma of the buccal mucosa (*n* = 1), squamous cell carcinoma of the upper jaw (*n* = 1), intraoral salivary duct carcinoma (*n* = 1), acinic cell carcinoma of the parotid gland (*n* = 1), adenocarcinoma of the palate (*n* = 1), intestinal-type adenocarcinoma of the sinonasal tract (*n* = 1), and synchronic squamous cell carcinoma of the upper jaw and of the floor of the mouth (*n* = 1). The mean interval between NR-CT and UHR-CT was 379 days (time range between 178 and 661 days). 

### 3.2. Subjective Image Quality

UHR-CT was significantly superior to NR-CT regarding subjective image quality for all defined parameters, particularly with great differences in image noise (UHR-CT: 5 [4.25–5] vs. NR-CT: 3 [2.0–3.0], *p* = 0.000) and image sharpness (UHR-CT: 5 [5.0–5.0] vs. NR-CT: 3.0 [3.0–3.0], *p* < 0.000). 

**Figure 4:**
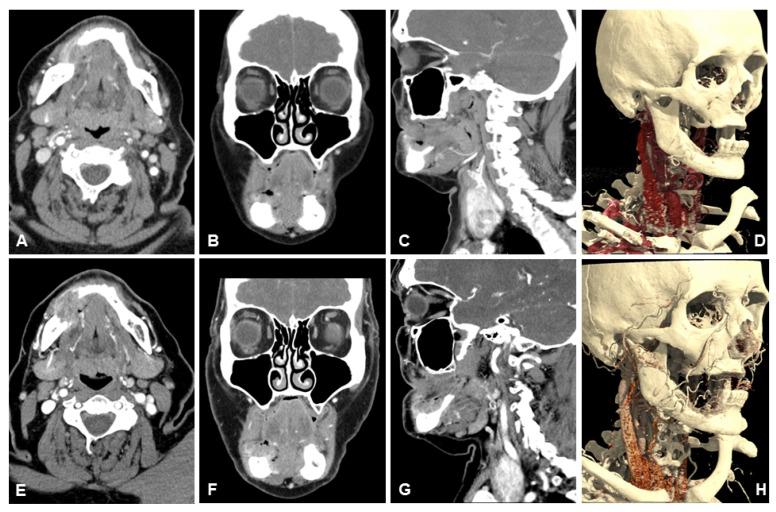
Example of a 79-year old male patient with a squamous cell carcinoma of the right lower jaw. Patient initially refused surgery, thus a 3-months follow-up examination was performed. Note the clear depiction and hypervascularization of the tumor margins and the lower noise of UHR-CT, especially in the axial plane. In the coronal reconstruction you can beautifully depict the tumor infiltration of the perimandibular fat and the contours of the sublingual space. (**A**) axial 3 mm reconstruction NR-CT, (**B**) sagittal 3 mm reconstruction NR-CT, (**C**) coronal 3 mm reconstruction NR-CT, (**D**) best possible 3D-reconstruction NR-CT, (**E**) axial 3 mm reconstruction UHR-CT, (**F**) sagittal 3 mm reconstruction UHR-CT, (**G**) coronal 3 mm reconstruction UHR-CT, and (**H**) best possible 3D-reconstruction UHR-CT.

**Figure 5:**
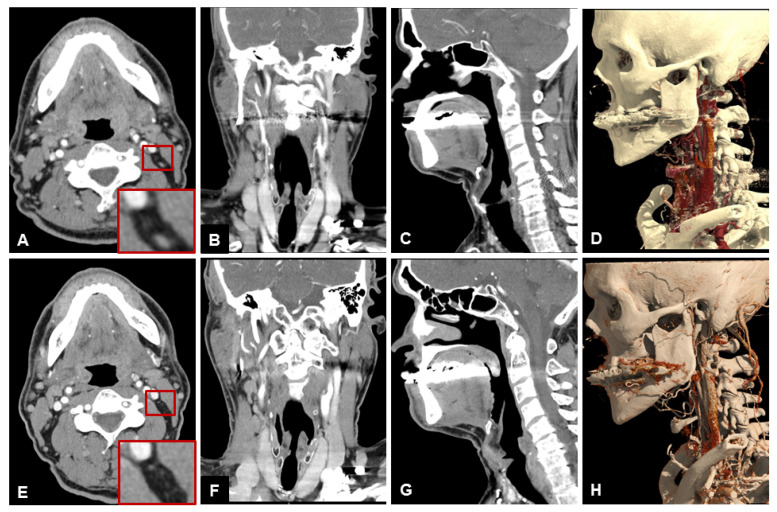
Example of a 57-year-old male patient with a squamous cell carcinoma of the lateral border of the tongue. Patient underwent initial resection. Both NR-CT and UHR-CT were performed after resection, and the carcinoma shows no signs of recurrence. Note the remarkably improved delineation of muscle and vessels with UHR-CT. (**A**) axial 3 mm reconstruction NR-CT, (**B**) coronal 3 mm reconstruction NR-CT, (**C**) sagittal 3 mm reconstruction NR-CT, (**D**) best possible 3D-reconstruction NR-CT, (**E**) axial 3 mm reconstruction UHR-CT, (**F**) coronal 3 mm reconstruction UHR-CT, (**G**) sagittal 3 mm reconstruction UHR-CT, (**H**) best possible 3D-reconstruction UHR-CT.

**Figure 6:**
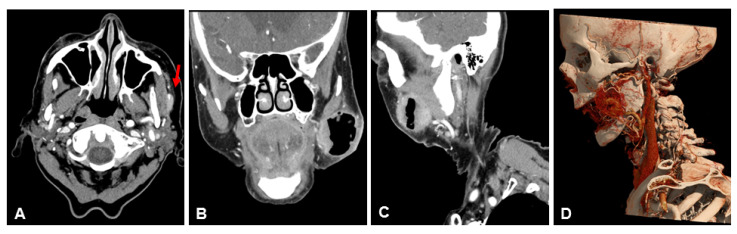
Example of a 93-year-old female patient with a squamous cell carcinoma of the left cheek. UHR-CT enables an accurate identification of arterial supply and venous drainage of the neoplasm, which is clearly depicted with 3D-reconstruction. It is possible to precisely differentiate between perineural tumor spread (red arrow) and adjacent muscle. (**A**) axial 3 mm reconstruction UHR-CT, (**B**) coronal 3 mm reconstruction UHR-CT, (**C**) sagittal 3 mm reconstruction UHR-CT, (**D**) best possible 3D-reconstruction NR-CT.

Table 3 lists the scores of both readers for subjective image quality in detail. Patient examples are given in Figure 4, Figure 5 and Figure 6.

Except for the skull-base and infratemporal fossa (ICC ≤ 0.6), the inter-rater agreement showed good to excellent values (ICC ≥ 0.7–1.0) for the UHR-CT images for all the defined parameters. Good to excellent inter-rater agreement could be demonstrated for all the defined parameters (ICC ≥ 0.82–0.99) when using NR-CT. All the ICCs are listed in Table 2.

### 3.3. Objective Image Quality

**Figure 7:**
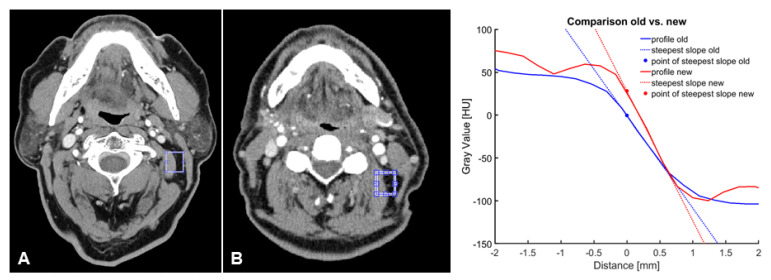
Comparison of contrast-to-noise-ratio of (**A**) UHR-CT and (**B**) NR-CT in a 57-year-old male patient with a squamous cell carcinoma of the left upper jaw.

**Figure 8:**
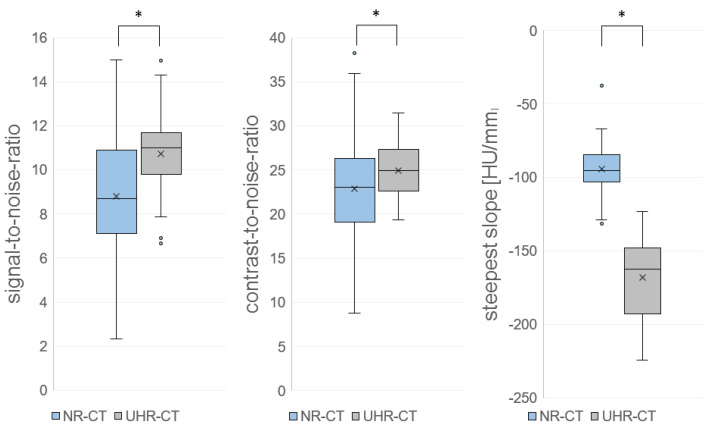
Boxplot of SNR and CNR of the quantitative analysis, as well as steepest slope [HU/mm] (NR-CT vs. UHR-CT). Asterisks indicates significanct effects.

The assessment of objective image criteria demonstrated that the UHR-CT images had significantly increased SNR and CNR values, as described in Table 4. As illustrated in Figure 7 and Figure 8, the contrast-to-noise ratio of both CT methods revealed that the steepness of the slope of gray-value transitions between fat and muscle tissue decreased from the NR-CT images: −94.5 ± −5.5 to in UHR-CT images: −168.4 ± −9.4 HU/mm (*p* < 0.0001). 

### 3.4. Radiation Dose

Dose exposure was evaluated by comparison of CTDIvol, DLP, and mean effective dose in millisievert (msv). The CTDIvol of the NR-CT was averaged at 14.0 ± 0.9 mGy and normalized DLP (approximated mean scan length of 25 cm) at 349.8 ± 23.7 mGy*cm. Dose exposure with UHR-CT was significantly lower with an average CTDIvol of 10.0 ± 0.7 mGy and a DLP of 250 ± 18.6 mGy*cm. Thereby, the UHR-CT led to a 29% reduction in the mean effective dose (NR-CT 2.03 ± 0.14; UHR-CT 1.45 ± 0.11 msv, *p* < 0.0001).

## 4. Discussion

This study aimed to investigate the potential benefits of a novel UHR-CT with a deep learning–based image reconstruction engine (AiCE) for head and neck imaging, as compared to prior NR-CT. Therefore, we evaluated subjective and objective image quality as well as radiation dose.

The results indicate that head and neck UHR-CT with AiCE yields excellent subjective and objective image quality and excellent depiction of tumorous lesions, superior to NR-CT. Data analysis revealed higher SNR and CNR and higher image sharpness for UHR-CT. Despite the increased spatial resolution and overall image quality, UHR-CT was associated with 29% lower radiation doses as compared to NR-CT. The radiation dose reduction may be mainly attributed to the new deep learning–based image reconstruction engine (AiCE) and also partially to the new UHR detector system that provides relatively low electronic noise [22,23,24].

During the last decade, IR algorithms have been established in clinical routine and enabled significant improvement in image quality and radiation dose reduction as compared to filtered back projection (FBP) [25,26,27,28]. Recently, various deep-learning algorithms have been introduced into clinical routine and may enable further reduction in radiation exposure as well as required iodine contrast as compared to IR [29,30,31,32]. AiCE, for instance, is trained to differentiate signal from noise and aims at reducing noise while improving low- contrast detectability and maintaining spatial resolution and image quality, thus enabling significant dose reduction and better image quality in comparison with plain IR [9,10,11]. 

To date, evidence of the additive value of UHR-CT remains to be elucidated, with few technical reports available [33,34,35,36,37]. In this study, by evaluating image quality and radiation dose of UHR-CT combined with AiCE in head and neck imaging, we were able to show for the first time that UHR-CT is significantly superior to NR-CT regarding subjective image quality for all the defined parameters. Particularly significant advantages in image noise, image sharpness, and diagnostic acceptability were observed. Thereby, UHR-CT demonstrated excellent soft tissue contrast and delineation. We used a slice thickness of 1 mm and 3 mm for subjective and objective image evaluation. Thereby, SNR and CNR will show higher results in 3 mm slice thickness, while sharpness and delineation of bone and vessels will improve using 3 mm slice thickness. Thus, slice thickness was consistently applied to compare subjective and objective image quality. 

Our findings may be of particular clinical relevance, as CT represents the method of choice in head and neck imaging. Since the structures of interest are usually small and some of these tumors tend to show perineural spread as well as skull base invasion, a high spatial resolution and good CNR are of utmost importance in the exact evaluation of the known predilection sites in order to enable early detection of clinically often silent complications [38,39,40]. Furthermore, with the changes in the 8th Edition of the American Joint Committee on Cancer (AJCC) of 2019, the appropriate assessment of tumor extension is of increasing importance for local tumor staging [1]. As shown previously, NR-CT can be used for the assessment of DOI but often results in overstaging [41,42,43]. Our subjective image analysis revealed that UHR-CT is significantly superior in the assessment of various highly relevant anatomic regions, such as the skull base, the infratemporal fossa, and the nasopharyngeal, oropharyngeal, and hypopharyngeal space. This is consistent with our clinical experience that in patients with certain head and neck pathologies, UHR-CT offers a chance for a more accurate tumor assessment.

In addition to the generation of ultra-high resolution CT images, the radiation dose decreased compared to NR-CT. DLP is defined as CTDIvol multiplied with scan length. Thus, for comparability and regarding our averaged scan length, DLP values for NR-CT and UHR-CT were normalized to a mean scan length of 25 cm. With a DLP of 250 ± 18.6 mGy*cm calculated with a CTDIvol of 10.0± 0.7 mGy, we managed to stay markedly below the currently updated diagnostic reference values for CT diagnostic in the head and neck region, published by the Federal Office for Radiation Protection (DLP of 285 [mGy*cm] calculated with a scan length of 19 cm and a CTDIvol of 15 mGy) [44].

Thus, patients will clearly benefit from this new technique of generating CT images with impressive image quality at comparatively low radiation doses. As CT is typically the first-line imaging tool, this may not only affect tumor assessment of the head and neck region but can be of high value in clinical emergencies with infectious diseases of the head and neck region [2,3].

This study has limitations. As it represents a single-centered, retrospective study, it is associated with selection bias. In order to reduce recall bias, the NR-CT and UHR-CT images were mixed and randomized and evaluated within a six-week time gap. Notably, both readers were uninformed that for each patient, both NR-CT and UHR-CT images were included. Nevertheless, as the image quality of the UHR-CT together with the AiCE reconstruction was substantially superior, the evaluating radiologists were still able to discriminate between the two groups. However, since the higher spatial resolution of UHR-CT results in greater image noise and thus generally requires a significantly increased dose for the same low-contrast detectability [8], we did not evaluate UHR-CT without AiCE and aimed at minimizing the applied radiation dose. Consequently, it is likely that both techniques contribute to improved image quality. UHR-CT results in a higher spatial resolution, while AiCE reduces noise and improves edge sharpness; hence we are unable discriminate the proportional impact of both techniques.

According to the German Guideline Program in Oncology, routine follow-up is performed semi-annually in the first two years after diagnosis and annually thereafter. As we evaluated clinically indicated routine head and neck CTs, the mean interval between the NR-CT and the follow-up UHR-CT was one year [45]. Thus, despite excluding patients with major anatomic changes in between, small anatomic changes or anatomical distortion due of scarring or weight change cannot be fully excluded. Furthermore, the small sample size does not allow generalization of our findings, and we did not evaluate the diagnostic accuracy for particular diseases. Our study was focused on image quality. We did not assess the further potential of AiCE for radiation dose reduction. Therefore, future prospective studies with larger sample sizes and homogeneous pathologies are needed.

## 5. Conclusions

Compared to NR-CT, UHR-CT combined with AiCE provides superior image quality at a markedly lower radiation dose. With improved soft tissue assessment and potentially improved tumor detection, UHR-CT may add further value to the role of CT in the assessment of head and neck pathologies.

## Figures and Tables

**Table 1:** Technical parameters of both CT scanners.

	NR-CT	UHR-CT
Focal spot size	0.4 × 0.5 mm	0.4 × 0.5 mm
Detector element size	1.6 × 1.4 mm	0.25 × 0.25 mm
Reconstruction Matrix	512	1024
Beam collimation	0.5 mm × 32	0.25 mm × 160
Pitch factor	0.8	0.569
Tube voltage	120 kV	120 kV
FOV	240 mm	240 mm

**Table 2:** Adapted Likert scale for subjective image evaluation.

Grading	Image Noise	Image Sharpness	Artifacts	Diagnostic Acceptability
1	Unacceptable	Blurry	Present and affecting image interpretation	Unacceptable
2	Increased	Poorer than average	Present and affecting visualization of normal structures	Suboptimal
3	Average	Subtle lesion	Present but not affecting visualization of normal structures	Average
4	Less than average	Clearly visualized lesion, poor margin	None	Above average
5	Minimum or no noise	Clearly visualized lesion, clearly visualized margin		excellent

**Table 3:** Comparison of subjective image quality between UHR-CT and NR-CT for both readers.

Assessed Parameter	UHR-CT Reader 1 (IQR)	UHR-CT Reader 2 (IQR)	ICC UHR-CT	NR-CT Reader 1 (IQR)	NR-CT Reader 2 (IQR)	ICC NR-CT	*p*-Value
Image noise	5 (4.25–5)	5 (5–5)	0.93	3 (2–3)	3 (2–3)	0.93	<0.000
Image sharpness	5 (5–5)	5 (5–5)	0.70	3 (2–3)	3 (2–3)	0.91	<0.000
Artifacts	1 (1–3)	1 (1–3)	1.00	1 (1–1)	1 (1–1)	0.99	<0.046
Diagnostic acceptability	4 (3–4.75)	4 (4–5)	0.91	2 (2–3)	2 (2–3)	0.96	<0.000
Skull base	5 (5–5)	5 (5–5)	0.60	3 (2–3)	3 (3–4)	0.80	<0.000
Infratemporal fossa	5 (5–5)	5 (5–5)	0.36	3 (3–3)	3 (3–3)	0.91	<0.000
Nasal cavity	5 (5–5)	5 (5–5)	0.85	3 (3–3)	3 (3–3)	0.95	<0.000
Paranasal sinuses	5 (5–5)	5 (5–5)	0.93	3 (3–4)	3 (3–4)	0.87	<0.000
Nasopharyngeal space	5 (5–5)	5 (4–5)	0.71	3 (3–3)	3 (3–3)	0.89	<0.000
Oropharyngeal space	4 (2–5)	4 (3–4.5)	0.97	2 (1–2)	2 (1–2)	0.98	<0.000
Hypopharyngeal space	5 (4–5)	5 (4–5)	0.91	3 (3–3)	3 (2.25–3)	0.91	<0.000
Oral cavity and buccal mucosa	2 (1–4)	2 (1–4)	0.99	1 (1–2)	1 (1–2)	0.97	<0.019
Floor of mouth	5 (4.25–5)	5 (4–5)	0.98	3 (3–3)	3 (2.25–3)	0.89	<0.000
Lymph nodes Level I	5 (5–5)	5 (5–5)	0.72	3 (3–3)	3 (3–3)	0.83	<0.000
Lymph nodes Level II-IV	5 (5–5)	5 (5–5)	0.91	3 (3–3)	3 (3–3)	0.83	<0.000
Jugular fossa	5 (4–5)	5 (5–5)	0.91	3 (3–3)	3 (3–3)	0.82	<0.000
Thyroid and upper mediastinum	5 (4.25–5)	5 (5–5)	0.91	3 (3–3)	3 (3–3)	0.84	<0.000
Salivary glands	4 (3–5)	4 (3–5)	0.94	2 (2–2.75)	2 (2–3)	0.86	<0.000
Carotid artery origin	5 (3.25–5)	5 (4–5)	0.89	3 (3–4)	3 (3–4)	0.88	<0.000
Vertebral artery V1	5 (3–5)	5 (4–5)	0.97	3 (3–4)	3 (2.25–4)	0.91	<0.000
Carotid artery bifurcation	5 (4–5)	5 (4–5)	0.97	3 (3–3.75)	3 (3–3.75)	0.96	<0.000
Vertebral artery V2	5 (4–5)	5 (4–5)	0.99	3 (2–3)	3 (2–3)	0.99	<0.000
Carotid artery C1/2	5 (5–5)	5 (4.25–5)	0.99	2 (2–3)	3 (2–3)	0.95	<0.000
Vertebral artery V3	5 (5–5)	5 (4.25–5)	0.97	2 (2–3)	2 (2–3)	0.96	<0.000

**Table 4:** Comparison of objective image quality between UHR-CT and NR-CT.

	UHR-CT	NR-CT	*p*-Value
SNR	10.8 [10.2–11.3]	8.8 [7.9–9.6]	<0.000
Steepest slope [HU/mm]	−168.4 [−(177.8–159.4)]	−94.5 [−(100.1–89.0)]	<0.000
Distance [mm] within the IQR	−0.56 [−(0.5–0.58)]	−0.97 [−(1.02–0.908)]	<0.000
CNR	26.1 [24.2–26.0]	22.9 [20.9–24.9]	<0.025

## Data Availability

Data are contained within the article.

## Abbreviations

AEC	auto-exposure control
AiCE	Advanced intelligent Clear-IQ Engine
CNR	contrast to noise-ratio
CT	computed tomography
CTDIvol	Volume computed tomography dose index
DLP	dose length product
FBP	filtered back-projection
FoV	field of view
ICC	intraclass correlation
IR	iterative reconstruction
KV	kilovolt
MBIR	statistical model based iterative reconstruction
MRI	Magnetic resonance imaging
mSv	millisievert
NR	normal resolution
SNR	signal to noise-ratio
UHR	ultra-high resolution
